# Resveratrol induces immunogenic cell death of human and murine ovarian carcinoma cells

**DOI:** 10.1186/s13027-019-0247-4

**Published:** 2019-10-18

**Authors:** Yanke Zhang, Sufen Yang, Yang Yang, Teng Liu

**Affiliations:** The Affiliated Hospital of Medical School of Ningbo University, No.247 Renmin Road, Jiangbei District, Ningbo, 315020 Zhejiang China

**Keywords:** Resveratrol, Ovarian carcinoma, Immunogenic cell death, Cytotoxic T cell

## Abstract

**Purpose:**

This study aimed to clarify whether immunogenic cell death (ICD) contributed to the anti-tumor action of resveratrol against ovarian carcinoma.

**Methods:**

Resveratrol suppressed cell proliferation and induced apoptosis in ovarian carcinoma cells. In addition, resveratrol treatment stimulated cell surface exposure of calreticulin, HMGB1 secretion and ATP release.

**Results:**

Vaccination with resveratrol-pretreated ID8 cells significantly inhibited growth of subsequent inoculated xenograft tumor. Direct administration with resveratrol suppressed tumor progression accompanied with compromised cell proliferation and enhanced cell apoptosis. We further characterized increases of both mature dendritic cells and cytotoxic T cells in xenograft tumor in response to resveratrol treatment, which also inhibited TGF-β production and stimulated both IL12p7 and IFN-γ secretion. Most importantly, we demonstrated that combination with PD-1 antibody greatly inhibited tumor growth, while depletion of CD8+ T cells by neutralizing antibody restored xenograft progression.

**Conclusion:**

Our data suggested resveratrol exerted anti-tumor action against ovarian cancer via both apoptosis and ICD pathways.

## Introduction

Ovarian carcinoma is one of three most common gynecologic malignancies with the highest mortality, and positions 5th with respect to cancer-related death among 10 major human cancers in women worldwide [1]. Currently, the primary option with curative purpose against ovarian cancer is surgical resection with adjuvant chemical drugs or radiotherapy [2]. However, in view of the intrinsic evident side effects, huge medical expenses and primary and acquired drug resistance, the clinical outcomes are far from satisfactory [3]. Therefore, exploitation of novel, low toxic, highly efficient anti-tumor drugs or chemotherapy re-sensitizing compounds is critical to the general improvement in terms of prognosis in ovarian cancer patients.

Resveratrol (RES) is non-flavonoid polyphenol containing stilbene structural elements, which has been widely identified in multiple plants including grapes and lilies [4]. The medicinal effects of RES have been documented in anti-inflammation, anti-microbial, anti-oxidant, antiviral, anti-atherosclerosis, immunomodulatory and neuroprotection. Most notably, accumulative evidences support significant anti-tumor properties of RES against diverse human cancers, and a number of signaling pathways have been unraveled mechanistically contributing to the anti-proliferative and apoptosis-inducing effects elicited by RES. The apparent therapeutic activities of RES have been displayed in hepatocellular carcinoma, ovarian carcinoma and leukemia. RES was first described to suppress hepatoma cell invasion independently of its anti-proliferative activities [5]. Opipari et al. reported RES induced autophagocytosis in ovarian cancer cells [6]. Jang et al. for the first-time uncovered cancer chemo-preventive properties of RES in promyelocytic leukemia cells [7]. The subsequent investigations elucidate the potential of RES in inducing cell apoptosis in ovarian cancer cells [8]. However, the possibility of RES in immune activation and promotion of immunogenic cell death (ICD) in this scenario is still to be defined.

Recently, emerging evidences disclosed that some platinum-based chemical drugs, such as oxaliplatin, provoked ICD in addition to cell apoptosis in cancer cells [9]. Generally, three major signal cues have been released when host cells experience ICD, including cell surface exposure of calreticulin (CRT) which stimulates phagocytosis of dendritic cells (DCs), ATP release which recruits DC cells, and secretion of High Mobility Group Box 1 (HMGB1) which promotes complexation of DC cells with moribund tumor cells and induces T cells-based specific tumor immunity [10]. ICD consequently elicits DC maturation and infiltration of cytotoxic T cell into tumor mass, which renders great opportunity for combinational therapies with immune checkpoint inhibitor such as PD-L1 and PD-1 antibodies [11].

In this study, we focused on the anti-tumor activities of RES in ovarian carcinoma, and particular attentions have been invested in the potential role of ICD both in vivo and in vitro. Co-employment of PD-1 antibody would provide experimental evidence in support of combination of ICD cue with immune checkpoint inhibitor, and pave the way for future clinical trials.

## Materials and methods

### Cell culture and treatment

Human ovarian carcinoma cell lines SKOV3 and A2780 and mouse ovarian carcinoma cell line ID8 were purchased from the Cell Bank of Chinese Academy of Sciences (Shanghai, China). Cell identities were authenticated by STR method. All cells were maintained in RPMI-1640 culture medium containing 15% fetal bovine serum plus 1% penicillin/streptomycin and cultured in humidified CO_2_ (5%) incubator. RES was purchased from Sigma-Aldrich (MO, USA) and dissolved in phosphate buffered saline (PBS) to prepare 100 mM stock solution.

### Proliferation assays

The cell proliferation was assessed by 3-(4,5-Dimethylthiazol-2-yl)-2,5-diphenyltetrazolium bromide (MTT) viability assays and colony formation assays in both SKOV3 and A2780 cells. For MTT assay, exponentially growing cells were seeded into 96-well plates (8000 cells /100 μl) and incubated at 37 °C in CO_2_ incubator for 24 h. The different concentrations of RES were then added and cells were continuously cultured for another 48 h. MTT stock solution was added to a final concentration of 1 g/L and followed by 2 h of incubation. 125 μL extraction buffer was added to each well and incubated overnight at 37 °C. OD 570 nm was measured using a microplate reader (Molecular Devices, CA, USA). For colony formation, both SKOV3 and A2780 cells were pre-treated with different doses of RES (25 and 50 μM) for 48 h and 1000 cells were seeded into each well of 6-well plate. Cells were maintained in CO_2_ incubator for 2 weeks. The formed colonies were fixed with ice-cold methanol and stained with 0.5% crystal violet (Sangon Biotech, Shanghai, China).

### Cell apoptosis

Cell death was determined by PI/Annexin V staining (BestBio, Shanghai, China). Both SKOV3 and A2780 cells were subjected to RES treatment as described previously and prepared into single-cell suspension in staining buffer containing PI and Annexin V. Staining was performed for 15 min at room temperature in dark, and apoptotic cells were then analyzed by flow cytometry (CytoFLEX, Beckman Coulter, CA, USA).

### Cell surface CRT analysis

SKOV3 and A2780 cells subjected to RES treatments were collected and residual culture medium was completely removed by PBS wash. Fixation was performed in 0.25% paraformaldehyde in PBS for 15 min and followed by primary antibody (FITC-CRT, Novusbio, CO, USA) incubation for 2 h at 4 °C. Alexa Fluor 488-conjugated secondary antibody (ThermoFisher, MA, USA) was subsequently applied for another 30 min. Samples were then analyzed with CytoFLEX flow cytometry (Beckman-Coulter, CA, USA) to quantify cell surface CRT.

### Detection of HMGB1 release

The culture medium was collected from RES-treated SKOV3 and A2780 cells and cell debris were completely discarded via centrifugation. Equal volume of supernatant was boiled with sodium dodecyl sulfate polyacrylamide gel electrophoresis loading buffer for 5 min and resolved by 12% gel. Protein samples were transferred onto polyvinylidene difluoride membrane and immunoblotted with anti-HMGB1 antibody (CST#3935, 1:1000, Cell Signaling Technology, MA, USA). Blots were visualized with enhanced chemiluminescence kit (ECL, Millipore, MA, USA) according to the manufacturer’s instruction. BSA was used as the external control.

### ATP release determination

10^5^ cells were seeded into 6-well plate and treated with indicated dosage of RES for 48 h and culture medium was collected. Cell debris was completely removed by high-speed centrifugation. ATP content was measured with the chemiluminescence ATP Determination Kit (Life technologies, MA, USA) according to the manufacturer’s manual. Briefly, each sample was added into reaction solution containing D-luciferin and luciferase without ATP and reaction was allowed for 30 min. The luminescence intensity was then recorded and ATP concentration was calculated based on the pre-plotted standard curve.

### Anti-tumor vaccination

ID8 cells were pre-treated with RES (control, 25 and 50 μM) for 24 h and prepared into single-cell suspension at concentration of 10^6^ cells/100 μL. 100 μL of cell suspension was then inoculated subcutaneously into the left lower flank of 6-week-old female C57BL/C mice (Vital River, Beijing, China), and 5 × 106 naïve ID8 cells were inoculated into the contralateral flank 7 days later. All animals were housed in SPF class environment, and all experiments were performed strictly in accordance with the NIH animal care and use guidelines. The animal related investigations were approved by the Institutional Ethics Committee of The Affiliated Hospital of Medical School of Ningbo University.

### Xenograft tumor

The xenograft tumor model was established by subcutaneous inoculation of ID8 cell into C57BL/C mice. Different doses of RES (vehicle, 50, 100 mg/kg body weight) were administrated via intraperitoneal injection every day after tumor volume reached 50 mm^3^. The tumor volumes were measured with digital caliper every 3 days up to 3 weeks and calculated according to the formula V = (1/2) × length × width^2^. Mice were sacrificed after 21 days and xenograft tumor was weighed.

### Immune cells infiltration in xenograft tumor

Tumor tissues were digested with collagenase IV (1 mg/mL) and single-cell suspensions were prepared accordingly. The mature DCs were stained with CD80/CD86 (gate in CD45 + CD11b + CD11c + cell population) and analyzed by CytoFLEX cytometry (Beckman-Coulter, CA, USA). The cytotoxic T cells were stained by CD8 in CD45 population.

### Enzyme-linked immunosorbent assay (ELISA)

Xenograft tumor tissues were digested with collagenase IV first and supernatant was collected for TGF-β, IL12p7 and IFN-γ measurement with ELISA kits (ThermoFisher, MA, USA) according to the manufacturer’s instruction. Briefly, 100 μL of standard and samples were added into each well and allowed for incubation at room temperature for 2 h. Solution was completely aspirated and diluted antibody was added and allowed for incubation for 1 h. After rigorous wash, 100 μL of horseradish peroxidase conjugate was added and incubated at room temperature for 30 min. Subsequently, 100 μL of chromogenic substrate was replaced and incubated at room temperature for 30 min, which was terminated by addition of 100 μL stop solution. Absorption at 450 nm was recorded for concentration determination and OD 550 nm as calibrating reference.

### Immunohistochemistry analysis

In order to determine the relative proliferative index in xenograft tumor in response to RES treatment, the tumor tissues were fixed in 10% formalin followed with paraffin embedding. 5 μm tissue sections were mounted on glass slides and briefly deparaffinized and rehydrated. Deparaffinized slides were boiled for 5 min in 0.01 M sodium citrate buffer (pH 6.0) in a pressure cooker for antigen retrieval. After wash with PBS containing 0.1% Tween-20 buffer, the slides were incubated with PCNA antibody at 4 °C overnight. The chromogenic reaction was performed with the SPlink Detection Kit (ZSGB-Bio, Beijing, China) following the manufacture’s recommendation. The intensity was scored by researcher blind to group assignment.

### Statistical analysis

Data processing and analysis were performed with PRISM 6.0 software (GraphPad, CA, USA). Student’s t-test or one-way ANOVA analysis was employed for comparison purpose and *p* value was calculated. A p value < 0.05 was considered significantly different.

## Results

### RES exhibits anti-proliferation activity and induces apoptosis in human ovarian carcinoma cells

**Fig. 1:**
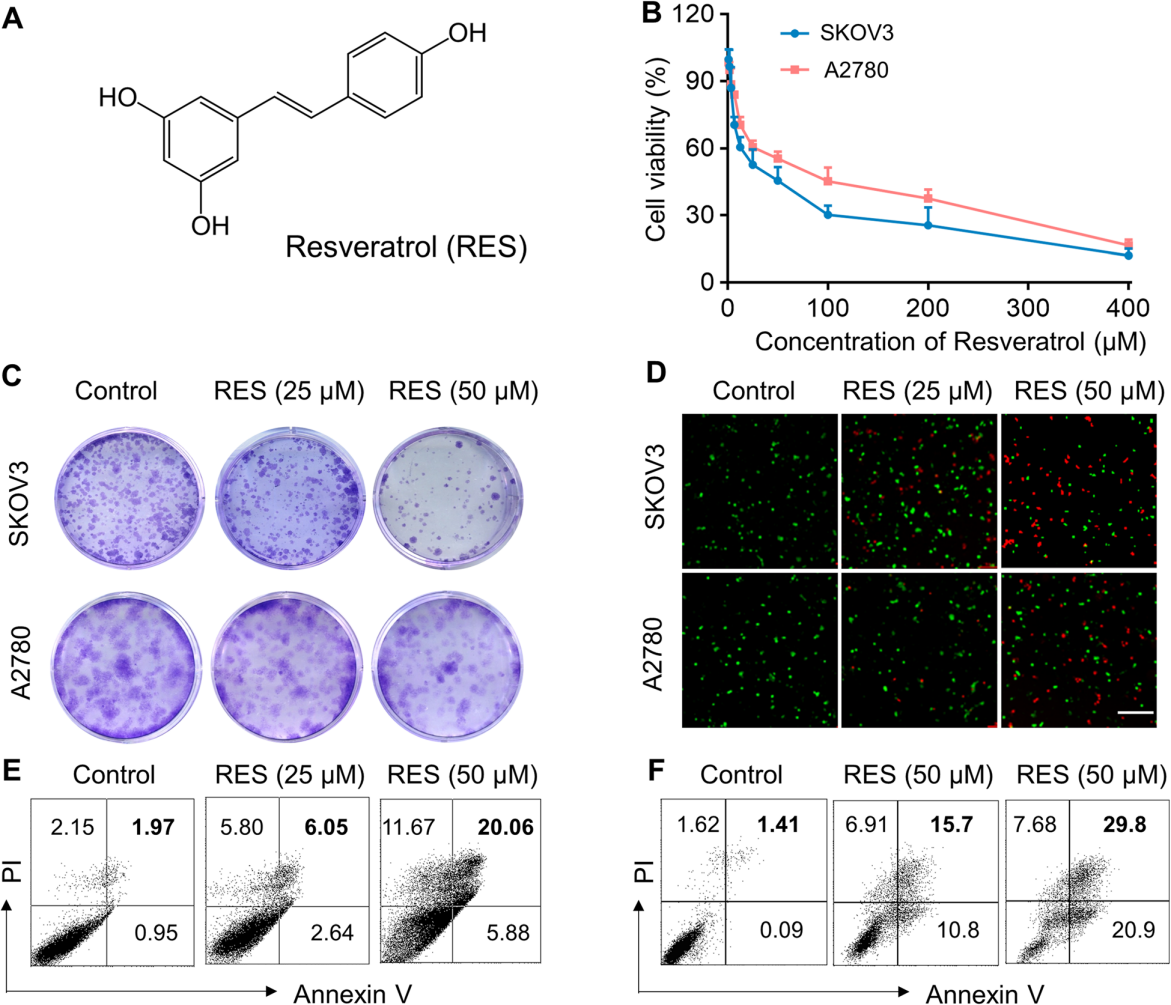
Resveratrol (RES) exhibits anti-proliferation activity and induces apoptosis in human ovarian carcinoma cells SKOV3 and A2780. **a** Chemical structure of resveratrol. **b** Dose-dependent killing of SKOV3 and A2780 cells by RES was determined by MTT assay. The cell viability was examined after 48 h incubation. **c** Colony formation ability of SKOV3 and A2780 cells after treated with RES (25 μM or 50 μM). Photographs of crystal violet-stained colonies are shown. **d** Fluorescence images of live/dead SKOV3 and A2780 cells after treated with different doses of RES. Cell viability was detected using LIVE/DEAD™ Viability/Cytotoxicity Kit. Live and dead cells were stained as green and red. Annexin V and PI staining by flow cytometric to analyze the percentages of apoptosis cells in SKOV3 cells (**e**) and A2780 cells (**f**) after treatment with different doses of RES

We first set out to evaluate the potential anti-tumor activities of RES against ovarian carcinoma in vitro. The molecular structure of RES is illustrated in Fig. 1a. Significant dose-dependent cytotoxicity of RES was observed in both SKOV3 and A2780 cells as indicated by MTT cell viability assay (Fig. 1b). Similarly, colony formation was greatly compromised by RES at either 25 μM or 50 μM in SKOV3 and A2780 cells, with the representative images provided in Fig. 1c. Cell apoptotic response to RES was further assessed, and the viable cells were tremendously decreased, as indicated by the green fluorescence accompanying with oppositely increase of dead cells indicated by redness (Fig. 1d). PI/Annexin staining results showed significant cell apoptosis in response to RES treatments in both SKOV3 and A2780 cell as well (Fig. 1e, f). Therefore, our data demonstrated that RES significantly inhibited cell proliferation and induced cell apoptosis in ovarian cancer cells in vitro.

### RES induces ICD in human ovarian carcinoma cells SKOV3 and A2780

**Fig. 2:**
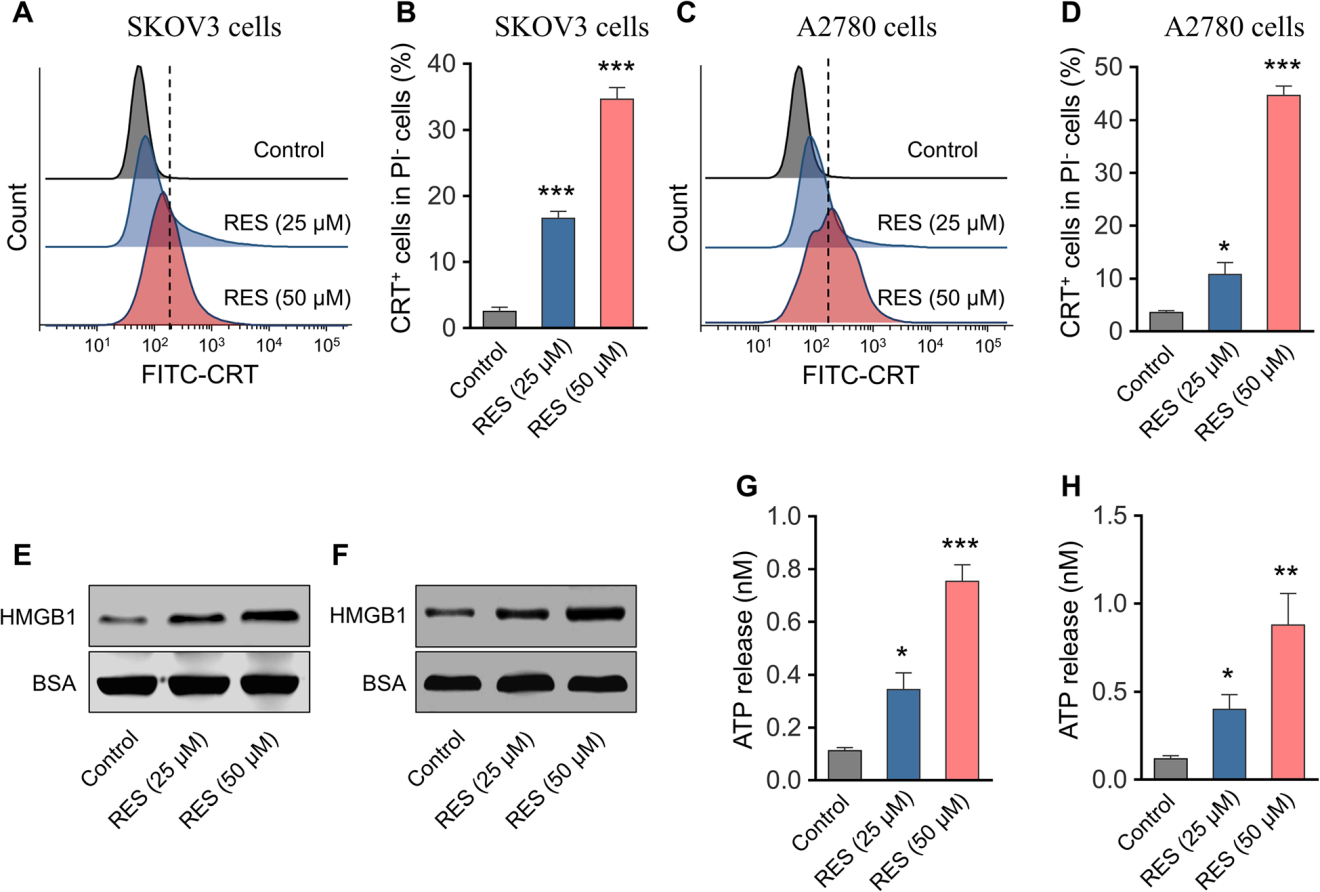
RES induces ICD in human ovarian carcinoma cells SKOV3 and A2780. **a** The surface exposure of calreticulin (CRT) of SKOV3 cells was determined by flow cytometry among viable (propidium iodine negative) cells after treated with RES (25 μM or 50 μM) for 24 h. Treated SKOV3 cells were stained with propidium iodine and FITC labeled anti-CRT antibodies according to the manufacturer’s instructions. **b** The percentage of CRT positive cells in PI negative cells was quantified based on the results of flow cytometry detection. Surface exposure of CRT (**c**) and percentage of CRT^+^ cells (**d**) in A2780 cells after RES treatment. Released HMGB1 in the medium supernatant of SKOV3 cells (**e**) and A2780 cells (**f**) treated with RES (25 μM or 50 μM) was measured by western blot, and BSA was used as the loading control. Amount of released ATP in the medium supernatant of SKOV3 cells (**g**) and A2780 cells (**h**) after RES treatment (25 μM or 50 μM) was determined by a chemiluminescent ATP Determination Kit. Data represent means ± SD. *p < 0.05, **p < 0.01, ***p < 0.001 (versus Control group)

Our preliminary data suggested the anti-tumor activities of RES against ovarian cancer cells in vitro through inhibition of cell proliferation and induction of cell apoptosis. Next, we sought to further determine whether RES stimulated ICD simultaneously in this scenario. The cell surface exposure of CRT was analyzed by flow cytometry in the viable cell population which was defined as PI-negative. As shown in Fig. 2a-d, RES treatment greatly increased cell surface CRT in both SKOV3 and A2780 cells. HMGB1 was markedly enriched in the supernatant from RES-treated SKOV3 and A2780 cells in comparison with control (Fig. 2e, f). We further quantified the released ATP in culture medium from either control or RES-treated cells by a chemiluminescent ATP determination kit. As shown in Fig. 2g and h, RES administration dramatically stimulated release of ATP in both cells as well. Taken together, our data uncovered that RES treatment induced ICD in human ovarian carcinoma cells, which consequently contributed to its anti-tumor properties.

### RES induces ICD in murine ovarian carcinoma cell ID8

**Fig. 3:**
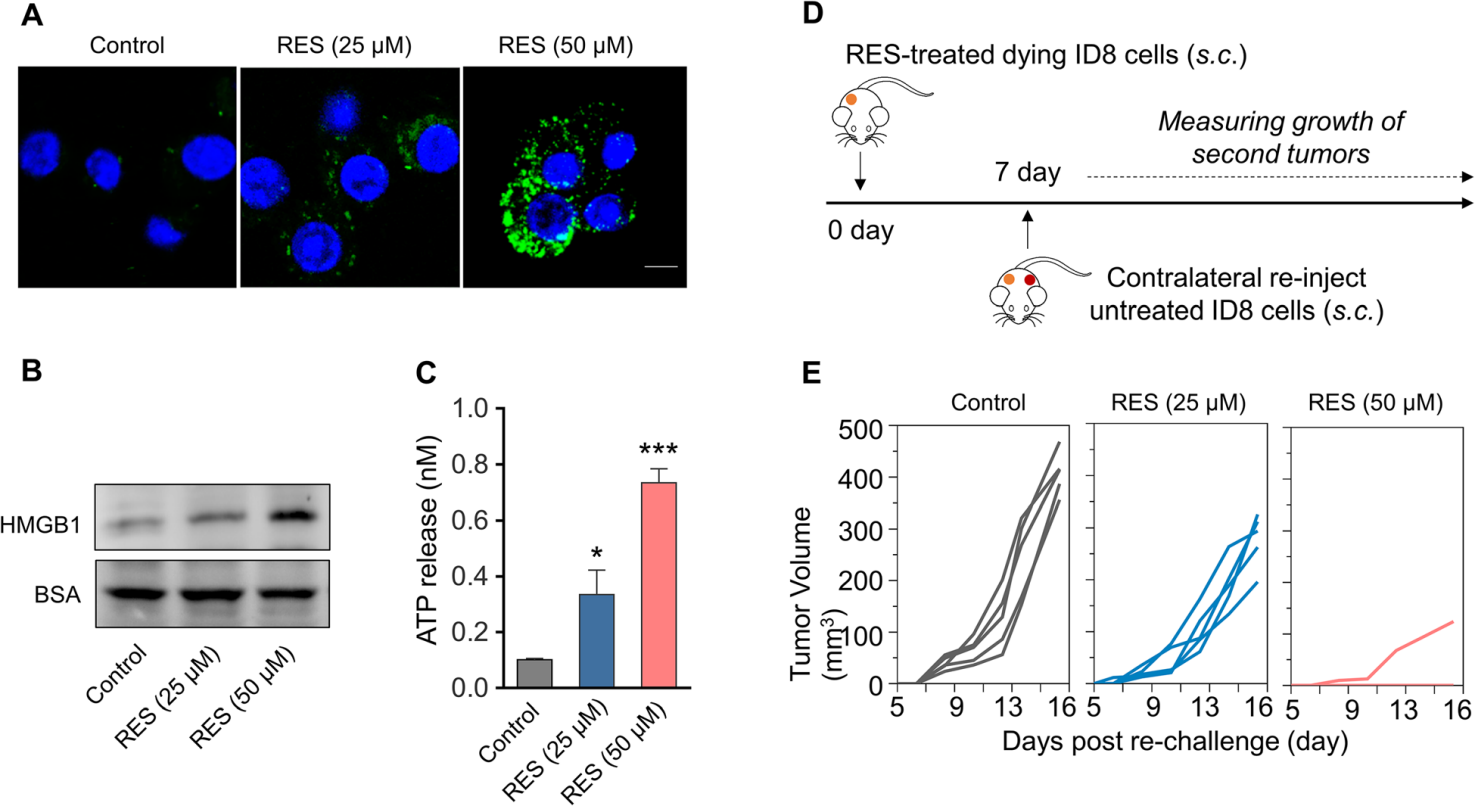
RES induces ICD in murine ovarian carcinoma cells ID8. **a** Fluorescent imaging of CRT exposed on surface of ID8 tumor cells after treated with RES (25 μM or 50 μM) for 24 h, and then cells were incubated with FITC-conjugated anti-calreticulin (FITC-anti-CRT) antibody for 2 h at 4 °C. After staining with DAPI, the cells were observed under confocal microscopy. **b** Released HMGB1 in the medium supernatant of ID8 cells treated with RES (25 μM or 50 μM) was measured by western blot, and BSA was used as the loading control. **c** Amount of released ATP in the medium supernatant of ID8 cells after RES treatment (25 μM or 50 μM) was determined by a chemiluminescent ATP Determination Kit. Data represent means ± SD. *p < 0.05, ***p < 0.001 (versus Control group). **d** Animal vaccination, using 2 rounds of subcutaneous (*s.c.*) injection of PBS or RES-treated 5*10^6^ dying ID cells 7 days apart, followed by *s.c.* injection of 5*10^6^ live cells on the contralateral side. Tumors growth were measured. **e** Growth of second tumors in animals vaccinated by dying tumor cells treated with RES or PBS

Next, we sought to consolidate ICD in response to RES treatment in vivo via employment of murine ovarian carcinoma cell ID8. Pre-treatment with RES (50 μM) significantly induced cell surface exposure of CRT in comparison with vehicle control as indicated by our immunofluorescence assay (Fig. 3a). RES treatment also induced increased release of HMGB1 (Fig. 3b) and ATP (Fig. 3c). The RES-treated immunogenic ID8 cells (1 × 10^6^ cells/100 μL) were subsequently injected subcutaneously into female C57BL/6 for vaccination purpose, and naïve ID8 cells (5 × 10^6^ cells/100 μL) were contralaterally inoculated 7 days post-vaccination, and growth was regularly monitored. The assay routine was illustrated in Fig. 3d. As shown in Fig. 3e, the xenograft tumor growth in RES-treated ID8 cell-pre-vaccinated group was significantly compromised, especially in the 50 μM RES group, in comparison with control. Our data unambiguously uncovered the immunogenic death elicited by RES treatment in animal model.

### In vivo anti-tumor effect of RES in ID8 tumor model

**Fig. 4:**
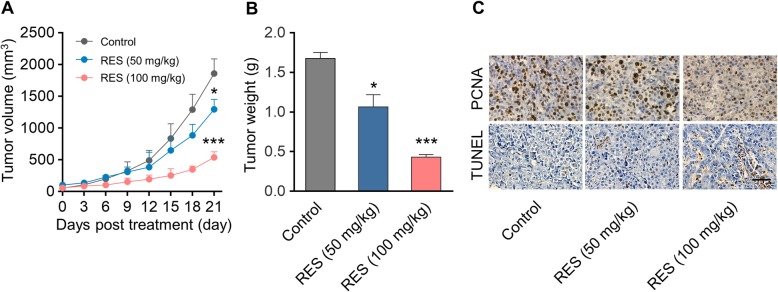
In vivo anti-tumor effect of RES in ID8 tumor model. **a** Growth curve of ID8 tumor volumes. Xenograft model of murine ovarian carcinoma was established by subcutaneous injection of ID8 cells on the flank of C57BL/6 mice (*n* = 8). Female C57BL/6 mice bearing ID8 tumors of ~ 50 mm^3^ were intraperitoneally (i.p.) injected with PBS or RES (50 or 100 mg per mouse body weight). Data represent means ± SD. *p < 0.05, ***p < 0.001 (versus Control group). **b** Weight of tumor tissues at the end of therapy. Data represent means ± SD. n = 8, *p < 0.05, ***p < 0.001 (versus Control group). **c** PCNA and TUNEL analysis of tumor tissues after treatment. The PCNA-positive proliferating cells and Ki67-positive apoptotic cells are stained brown. Scale bar was 20 μm

The direct anti-tumor properties of RES against ovarian carcinoma was further characterized in ID8 xenograft mouse model, which was established by subcutaneous injection of ID8 on the flank of C56BL/6 female mice. RES (50 or 100 mg/kg body weight) or PBS vehicle control was intraperitoneally administrated when xenograft tumor became palpable and volume approached 50 mm^3^. As shown in Fig. 4a, tumor progression was greatly repressed by RES administration compared with PBS group. Consequently, tumor weight from RES (100 mg/kg) group was dramatically decreased as well (Fig. 4b). The immunohistochemical results demonstrated evidently reduced cell proliferative index as indicated by PCNA with concomitantly increased cell apoptotic index as indicated by terminal deoxynucleotidyl transferase dUTP nick end labeling intensity in the RES-treated mice in comparison with control group (Fig. 4c). Our data provided evidence in support of the potential therapeutic value of RES in ovarian carcinoma with mechanistic inhibition of proliferation and promotion of apoptosis.

### RES treatment modulates the tumor immune microenvironment in the ID8 tumor model

**Fig. 5:**
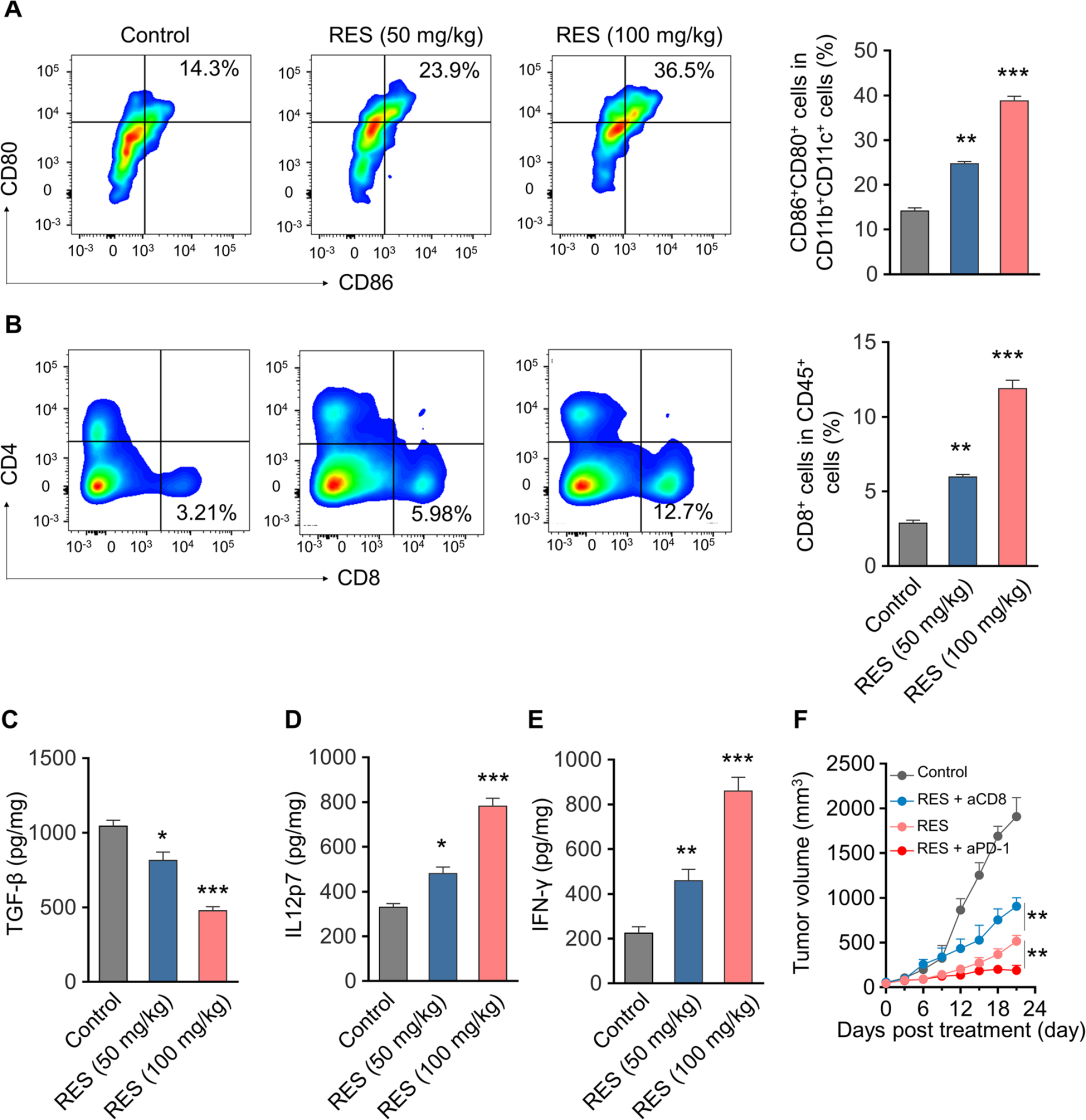
RES treatment modulates the tumor immune microenvironment in the ID8 tumor model. **a** Flow cytometry gating and histogram analysis of matured dendritic cells (DCs) in the tumor tissues at the end of treatment. The matured DCs were denoted as CD80^+^CD86^+^ populations (gate in CD45^+^CD11b^+^CD11c^+^ cell population). Data represent means ± SD, n = 8, **p < 0.01, ***p < 0.001, versus Control group; (**b**) Flow cytometry gating and histogram analysis of cytotoxic T cells (CD8^+^ T cells) in the CD45^+^ tumor infiltrating immune cells. Data represent means ± SD., n = 8, **p < 0.01, ***p < 0.001, versus Control group; ELISA results of TGF-β (**c**), IL12p70 (**d**) and IFN-γ (**e**) production in the tumors from mice receiving PBS or RES treatments. Data represent means ± SD. n = 8, *p < 0.05, **p < 0.01 ***p < 0.001, versus Control group. **f** Antitumor effect of combination of RES and aCD8 or aPD-1. To demonstrate the critical role of cytotoxic CD8^+^ T cells in antitumor immunity, anti-CD8 monoclonal antibody was i.p. injected in treatment group 3 days prior to the first drug administration and repeated every 3 days until the termination of the study, and anti-PD1 monoclonal antibody was i.p. injected in treatment group 7 days post first injection, and repeat 3 times. The injection dosages of aCD8 and aPD-1 were 200 μg/mouse and 100 μg/mouse, respectively. Data represent means ± SD. *n* = 8, **p < 0.01

To elucidate the immunologic mechanisms underlying the anti-tumor activities of RES, we further analyzed both mature DCs and cytotoxic T cells in the tumor tissues at the end point of drug treatment. As shown in Fig. 5a, the DC population as denoted as CD80 + CD86+ was significantly induced by RES treatment (100 mg/kg). Similarly, the cytotoxic T cells as indicated by CD8 were tremendously stimulated by RES administration in comparison with vehicle treatment (Fig. 5b, 100 mg/kg). Cytokines including TGF-β, IL12p7 and IFN-γ in the xenograft tumor tissues were determined by ELISA as well. Consistent with observed anti-tumoral properties of RES, TGF-β (Fig. 5c, *p* < 0.001) content was greatly decreased while both IL12p7 (Fig. 5d, *p* < 0.001) and IFN-γ (Fig. 5e, *p* < 0.001) were significantly induced by RES treatment compared with vehicle control. The critical contribution of T cells in elimination of tumor cells were further characterized by employment of either anti-CD8 or PD-1 antibodies. CD8-depletion greatly restored the tumor progression under treatment with RES, while co-administration with checkpoint inhibitor PD-1 antibody almost completely suppressed xenograft tumor growth (Fig. 5f). Our results suggested that RES treatment significantly modulated the immune microenvironment via induction of both mature DCs and cytotoxic T cells.

## Discussion

The anti-tumor properties of RES have been well-documented in a variety of human malignancies including hepatocellular carcinoma, ovarian carcinoma and leukemia. Liu et al. reported that RES inhibited cell proliferation and stimulated cell apoptosis in ovarian cancer cell through blockade of glycolysis and inhibition of AMPK/mTOR signaling [12]. In view of the acknowledged roles of RES in immunomodulation in multiple diseases, whether RES stimulated immune activation to contribute tumor killing actions was still be to defined. Here we first confirmed the anti-proliferative and apoptosis-inducing effects of RES against ovarian carcinoma cells in vitro, which was consistent with previous report. Furthermore, we demonstrated that RES treatment significantly provoked cell surface exposure of CRT protein, secretion of HMGB1 and ATP release, which suggested potential ICD in this scenario which was distinct to canonical apoptosis featured in clean, neat and poor ICD processing. To experimentally test this hypothesis in vivo, in this study we employed murine ovarian carcinoma cell line ID8, which was pre-treated with RES and cell surface exposure of CRT was characterized. Vaccination with RES-pretreated ID8 greatly suppressed the growth of xenograft tumor subsequently inoculated, which clearly indicated immune activation by RES treatment. The anti-tumor activities of RES was further demonstrated with naïve ID8 cell subcutaneous mouse model, wherein intraperitoneal administration with RES significantly inhibited tumor progression, which was accompanied by decreased cell proliferation and increased cell apoptosis. We further characterized the immune microenvironment in tumor tissues in response to RES treatment, and found evident increases in terms of both mature DCs and cytotoxic T cells. The secretion of TGF-β was remarkably decreased and both of IL12p7 and IFN-γ were induced as well. Most notably, combination with immune checkpoint inhibitor PD-1 antibody with RES markedly suppressed xenograft tumor growth, whereas depletion of CD8+ cell partially restored the tumor progression which was compromised by RES treatment alone. Taken together, our data suggested that RES induced apoptotic and ICD in ovarian carcinoma both in vitro and in vivo, which warranted further mechanistic investigations and clinical trials.

In contrast to conventional chemotherapy or immunotherapy alone, here we provided a novel paradigm to combine immunotherapeutic antibody with chemical drug with potential for immunogenicity for ovarian carcinoma management clinically. With prior RES treatment, the enhanced mature DCs and cytotoxic T cells significantly facilitated the anti-tumor actions of PD-1 antibody and potentiated therapeutic outcomes. In agreement with our findings, multiple investigations have previously addressed ICD in other tumor types. For example, Liu et al. reported the crizotinib-provoked ICD in non-small cell lung cancer [13]. Deng et al. suggested that combinational administration with tumor and its microenvironment dual targeting chemotherapy plus focal immune adjuvant therapy exerted effectively antitumor immunity in breast cancer [14]. Wan et al. demonstrated the improved cancer immunochemotherapy by dual functional immunostimulatory polymeric prodrug carrier with pendent indoximod [15]. Yuan et al. proposed that naphthyl quinoxaline thymidine conjugate as a potent antitumor agent after UV activation and elicited remarkable tumor suppressive effect through vaccination [16]. Yang et al. further provided evidences that through improved antitumor potency of thymidine conjugate, cRGD targeting liposome delivery system enhanced ICD under UV activation and functioned as a cancer vaccine [17]. In this regard, our results uncovered the vaccination potential of RES-pretreated cells and presented ICD response upon RES challenge, which rendered great potentials of RES for adjuvant administration with immunotherapies.

Noting worthily, our data highlighted the importance of both mature DCs and cytotoxic T cells underlying ICD elicited by RES, particularly underlined the consequent contributions of CD8+ T cells in this scenario. Depletion of CD8+ cells by neutralizing antibody led to greatly restoration of xenograft tumor progression even under RES treatment. The immunomodulatory actions of RES in tumor microenvironment resembled several reports addressing an array of chemical drugs in ovarian carcinoma. For instance, Napoletano et al. showed that bevacizumab-based chemotherapy elicited immunological effects against responding multi-treated recurrent ovarian tumor patients through facilitating recruitment of effector T cell subpopulations [18]. Mesnage et al. demonstrated that neoadjuvant chemotherapy promoted immune infiltration and PD-L1 expression in epithelial ovarian cancer [19]. Kodumudi et al. reported a novel chemo-immuno-modulating property of docetaxel by suppression of myeloid-derived suppressor cells in xenograft tumor bearing mice [20]. Zhang et al. proposed differential impairment of regulatory T cells other than effector T cells by paclitaxel-based chemotherapy [21]. These studies represented a promising direction for exploitation of novel chemicals with potential to improve the efficacy of immunotherapy clinically.

## Conclusion

In summary, we have uncovered that RES exerts anti-tumor activities in ovarian carcinoma via inducing ICD in addition to cell apoptosis, which holds great promise for combinational application with immunotherapy for clinical purpose.

## Data Availability

All data generated or analyzed during this study are included in this published in this article.

## References

[1] Torre LA, Trabert B, DeSantis CE, Miller KD, Samimi G, Runowicz CD, Gaudet MM, Jemal A, Siegel RL (2018). Ovarian cancer statistics, 2018. CA Cancer J Clin.

[2] Fujiwara Keiichi, McAlpine Jessica N., Lheureux Stephanie, Matsumura Noriomi, Oza Amit M. (2016). Paradigm Shift in the Management Strategy for Epithelial Ovarian Cancer. American Society of Clinical Oncology Educational Book.

[3] Agarwal R, Kaye SB (2003). Ovarian cancer: strategies for overcoming resistance to chemotherapy. Nat Rev Cancer.

[4] Nawaz Waqas, Zhou Zhongqin, Deng Sa, Ma Xiaodong, Ma Xiaochi, Li Chuangang, Shu Xiaohong (2017). Therapeutic Versatility of Resveratrol Derivatives. Nutrients.

[5] Kozuki Y, Miura Y, Yagasaki K (2001). Resveratrol suppresses hepatoma cell invasion independently of its anti-proliferative action. Cancer Lett.

[6] Opipari AW, Tan L, Boitano AE, Sorenson DR, Aurora A, Liu JR (2004). Resveratrol-induced autophagocytosis in ovarian cancer cells. Cancer Res.

[7] Jang M, Cai L, Udeani GO, Slowing KV, Thomas CF, Beecher CW, Fong HH, Farnsworth NR, Kinghorn AD, Mehta RG, Moon RC, Pezzuto JM (1997). Cancer chemopreventive activity of resveratrol, a natural product derived from grapes. Science.

[8] Gwak H, Kim S, Dhanasekaran DN, Song YS (2016). Resveratrol triggers ER stress-mediated apoptosis by disrupting N-linked glycosylation of proteins in ovarian cancer cells. Cancer Lett.

[9] Garg AD, More S, Rufo N, Mece O, Sassano ML, Agostinis P, Zitvogel L, Kroemer G, Galluzzi L (2017). Trial watch: immunogenic cell death induction by anticancer chemotherapeutics. Oncoimmunology.

[10] Showalter A, Limaye A, Oyer JL, Igarashi R, Kittipatarin C, Copik AJ, Khaled AR (2017). Cytokines in immunogenic cell death: applications for cancer immunotherapy. Cytokine.

[11] Pfirschke C, Engblom C, Rickelt S, Cortez-Retamozo V, Garris C, Pucci F, Yamazaki T, Poirier-Colame V, Newton A, Redouane Y, Lin YJ, Wojtkiewicz G, Iwamoto Y, Mino-Kenudson M, Huynh TG, Hynes RO, Freeman GJ, Kroemer G, Zitvogel L, Weissleder R, Pittet MJ (2016). Immunogenic chemotherapy sensitizes tumors to checkpoint blockade therapy. Immunity.

[12] Liu Y, Tong L, Luo Y, Li X, Chen G, Wang Y (2018). Resveratrol inhibits the proliferation and induces the apoptosis in ovarian cancer cells via inhibiting glycolysis and targeting AMPK/mTOR signaling pathway. J Cell Biochem.

[13] Liu P, Zhao L, Pol J, Levesque S, Petrazzuolo A, Pfirschke C, Engblom C, Rickelt S, Yamazaki T, Iribarren K, Senovilla L, Bezu L, Vacchelli E, Sica V, Melis A, Martin T, Lin X, Yang H, Li Q, Chen J, Durand S, Aprahamian F, Lefevre D, Broutin S, Paci A, Bongers A, Minard-Colin V, Tartour E, Zitvogel L, Apetoh L, Ma Y, Pittet MJ, Kepp O, Kroemer G (2019). Crizotinib-induced immunogenic cell death in non-small cell lung cancer. Nat Commun.

[14] Deng C, Zhang Q, Jia M, Zhao J, Sun X, Gong T, Zhang Z (2019). Tumors and their microenvironment dual-targeting chemotherapy with local immune adjuvant therapy for effective antitumor immunity against breast Cancer. Adv Sci (Weinh).

[15] Wan Zhuoya, Sun Jingjing, Xu Jieni, Moharil Pearl, Chen Jing, Xu Junchi, Zhu Junjie, Li Jiang, Huang Yixian, Xu Pengfei, Ma Xiaochao, Xie Wen, Lu Binfeng, Li Song (2019). Dual functional immunostimulatory polymeric prodrug carrier with pendent indoximod for enhanced cancer immunochemotherapy. Acta Biomaterialia.

[16] Yuan Y, Wang Z, Yang R, Qian T, Zhou Q (2019). Naphthyl quinoxaline thymidine conjugate is a potent anticancer agent post UVA activation and elicits marked inhibition of tumor growth through vaccination. Eur J Med Chem.

[17] Yang R, Wang Z, Yuan Y, Qian T, Zhou Q (2019). cRGD target liposome delivery system promoted immunogenic cell death through enhanced anticancer potency of a thymidine conjugate under UVA activation as a cancer vaccine. Eur J Med Chem.

[18] Napoletano Chiara, Ruscito Ilary, Bellati Filippo, Zizzari Ilaria Grazia, Rahimi Hassan, Gasparri Maria Luisa, Antonilli Morena, Panici Pierluigi Benedetti, Rughetti Aurelia, Nuti Marianna (2019). Bevacizumab-Based Chemotherapy Triggers Immunological Effects in Responding Multi-Treated Recurrent Ovarian Cancer Patients by Favoring the Recruitment of Effector T Cell Subsets. Journal of Clinical Medicine.

[19] Mesnage SJL, Auguste A, Genestie C, Dunant A, Pain E, Drusch F, Gouy S, Morice P, Bentivegna E, Lhomme C, Pautier P, Michels J, Le Formal A, Cheaib B, Adam J, Leary AF (2017). Neoadjuvant chemotherapy (NACT) increases immune infiltration and programmed death-ligand 1 (PD-L1) expression in epithelial ovarian cancer (EOC). Ann Oncol.

[20] Kodumudi KN, Woan K, Gilvary DL, Sahakian E, Wei S, Djeu JY (2010). A novel chemoimmunomodulating property of docetaxel: suppression of myeloid-derived suppressor cells in tumor bearers. Clin Cancer Res.

[21] Zhang L, Dermawan K, Jin M, Liu R, Zheng H, Xu L, Zhang Y, Cai Y, Chu Y, Xiong S (2008). Differential impairment of regulatory T cells rather than effector T cells by paclitaxel-based chemotherapy. Clin Immunol.

